# Cardiovascular risk in chronic kidney disease patients: intima-media thickness predicts the incidence and severity of histologically assessed medial calcification in radial arteries

**DOI:** 10.1186/s12882-015-0067-8

**Published:** 2015-06-03

**Authors:** Katarzyna Janda, Marcin Krzanowski, Mariusz Gajda, Paulina Dumnicka, Danuta Fedak, Grzegorz J. Lis, Piotr Jaśkowski, Agata Pietrzycka, Jan A. Litwin, Władysław Sułowicz

**Affiliations:** Chair and Department of Nephrology, Jagiellonian University, Medical College, Kopernika 15c, 31-501 Cracow, Poland; Chair and Department of Histology, Jagiellonian University, Medical College, Cracow, Poland; Department of Medical Diagnostics, Jagiellonian University, Medical College, Cracow, Poland; Chair of Clinical Biochemistry, Jagiellonian University, Medical College, Cracow, Poland; Radioligand Laboratory, Faculty of Pharmacy, Jagiellonian University, Medical College, Cracow, Poland

**Keywords:** Alizarin red staining, Calcification, Chronic kidney disease, Common carotid artery intima-media thickness, Radial artery

## Abstract

**Background:**

The objective of the study was to determine the relationship between common carotid artery intima-media thickness (CCA-IMT) and histologically assessed calcification of radial artery in relation to clinical features and laboratory markers of bone and mineral metabolism, inflammation, and oxidative stress in patients with stage 5 chronic kidney disease (CKD).

**Methods:**

The study comprised 59 patients (36 hemodialyzed, 23 predialysis). CCA-IMT was measured by ultrasonography; the biochemical parameters examined were assessed using routine laboratory methods, ELISA micro-plate immunoassays and spectrophotometry. Fragments of radial artery obtained during creation of hemodialysis access were cryosectioned and stained for calcifications using von Kossa method and alizarin red.

**Results:**

Glucose, osteoprotegerin, pentraxin 3 and Framingham risk score significantly correlated with CCA-IMT. In multiple regression analysis, OPG positively predicted CCA-IMT. Radial artery calcifications were found in 34 patients who showed higher CCA-IMT (0.98 ± 0.13 vs 0.86 ± 0.14 mm; *P* = 0.006). Higher CCA-IMT values were also associated with more advanced calcifications. CCA-IMT and the presence of plaques in common carotid artery were positive predictors of radial artery calcifications, independent of dialysis status, Framingham risk score, CRP and Ca x Pi [OR for calcifications 2.19 (1.08-4.45) per 0.1 mm increase in CCA-IMT]. The presence of radial artery calcifications was a significant predictor of mortality, independent of dialysis status and Framingham risk score [HR 3.16 (1.03-9.64)].

**Conclusions:**

In CKD patients, CCA-IMT examination can be used as a surrogate measure to assess the incidence and severity of arterial medial calcification which is associated with poor clinical outcome in these patients.

## Background

Vascular calcification, common carotid intima media thickness (CCA-IMT) and the presence of carotid plaques are strongly associated with cardiovascular disease in chronic kidney disease (CKD) patients [1, 2]. Arterial thickening contributes to elevated risk of cardiovascular episodes in patients on maintenance renal replacement therapy. The CCA-IMT, an early marker of vascular pathology, is commonly used to assess the advancement of atherosclerosis [3, 4]. In CKD patients, arterial calcifications occur in two distinct locations: in the tunica intima and in the tunica media [5–7]. Intimal calcification is observed in atherosclerosis and is associated with inflammatory infiltration and stenotic arterial lesions. Whereas atherosclerosis underlies ischemic heart disease and stroke, medial arterial calcification (MAC) decreases arterial compliance and increases arterial stiffening potentially leading to hypertension, left ventricular hypertrophy and congestive heart failure [8]. Moreover, in CKD, MAC also occurs in patients with advanced age and diabetes [5]. Coronary artery calcifications occur in about 50 % of CKD patients not yet on dialysis [9] and in 70–90 % of prevalent dialysis patients [10, 11].

The diagnostic procedures aimed at detection of medial calcification in patients are limited because non-invasive imaging techniques cannot reliably distinguish it from intimal calcification associated with atherosclerosis. Generally, arterial calcification can be detected by plain radiographs, computed tomography methods including electron-beam computed tomography (EBCT) and multi-slice (spiral) computed tomography (MSCT), as well as by ultrasonography [12, 13].

However, only histological assessment of the vessel differentiates changes located in the intima and in the media. The aim of the present study was to determine whether histologically assessed medial arterial calcification is significantly associated with higher CCA-IMT values and more prevalent cardiovascular events in patients with stage 5 chronic kidney disease. Moreover, we examined association between CCA-IMT and vascular calcification in relation to laboratory markers of bone and mineral metabolism, inflammation, and oxidative stress. We used small samples of radial artery wall obtained intravitally during creation of the arteriovenous fistula for hemodialysis access.

## Methods

### Study design

The study included patients with stage 5 CKD in whom arteriovenous fistula was created for the first time for hemodialysis access, allowing collection of radial artery samples for histological examination. Cross-sectional data were obtained immediately before that procedure, and included clinical assessment of patients, CCA-IMT measurements and assessment of laboratory parameters (markers of inflammation, calcification and oxidative stress). Longitudinal data were collected over a period of 3 following years and included the dates of renal transplantation and the dates and causes of death.

### Patients

The study population consisted of 59 consecutive patients (stage 5 of CKD), including 36 on maintenance hemodialysis (HD) and 23 predialysis. The majority of the subjects were men (38, i.e. 64 %). The mean age at the beginning of the study was 61 ± 16 years. Ten-year risk of coronary artery disease (CAD) was calculated by the Framingham Risk Score (FRS) in accordance with the published guidelines [14].

The intima-media thickness of the common carotid artery trunk (CCA-IMT) was assessed by ultrasonography (B presentation, Acuson 128 XP/10 apparatus equipped with linear head at 5/7 MHz) at the beginning of the study. The measurements were performed bilaterally at 0.5 cm and 2 cm below the division of the common carotid artery during diastolic phase of the heart cycle. The results were expressed as the arithmetic means of the values obtained for the left and right arteries.

The data on mortality was collected over a period of three years following creation of arteriovenous fistula for hemodialysis access. All deaths occurred in the hospital, therefore the dates and causes of death were determined on the basis of medical history documentation. The person who collected follow-up data was blind to the results of cross-sectional part of the study.

The study was approved by the Bioethics Committee of the Jagiellonian University and all patients signed an informed consent for their participation.

### Laboratory tests

In all patients, selected biochemical parameters were measured, including serum concentrations of creatinine, glucose, parathyroid hormone (iPTH), total calcium (Ca) and phosphate (Pi), inflammatory markers: high-sensitivity C-reactive protein (hsCRP), interleukin-6 (IL-6), pentraxin 3 (PTX3), and circulating calcification markers: osteopontin (OPN), osteoprotegerin (OPG), osteocalcin (OC), fibroblast growth factor 23 (FGF-23) and fetuin A. Oxidative stress was assessed by measuring ferric reducing ability of plasma (FRAP), 2,2-diphenyl-1-picrylhydrazyl (DPPH) scavenging and ferric reducing ability of ascorbate in plasma (FRASC). *Homeostasis Model of Assessment - Insulin Resistance* (HOMA-IR) was calculated by application of the international formula: fasting insulin (μIU/ml) × fasting glucose (mmol/l)/22.5. The estimated glomerular filtration rate (eGFR) was calculated by *Modification of Diet in Renal Disease* (MDRD) formula: eGFR = [186 × serum creatinine (umol/l) × 0.0113]^-1.154^ × age^-0.203^ × 114 × (0.742 for women) [15].

Blood samples of the patients were obtained at the beginning of the study, on the morning before creation of arteriovenous fistula for hemodialysis access. Serum samples for ELISA tests were aliquoted and stored at −70 °C until assayed (no longer than 3 months). Plasma samples used to assess oxidative stress parameters were protected from light, placed on ice and centrifuged within 2 h after collection, then aliquoted and stored at −30 °C until analysis (no longer than one month).

Routine biochemical tests were carried out using automatic biochemical analyzers: Hitachi 917 (Hitachi, Japan) and Modular P (Roche Diagnostics, Mannheim, Germany). Concentrations of CRP were measured using immunonephelometric method (Nephelometer BN II, Siemens Healthcare Diagnostics, Germany).

Inflammatory and calcification markers were assessed using ELISA micro-plate immunoassays and ELX808 automatic reader (BIO-TEK® Instruments Inc., Vermont, VT, USA). The following kits were applied: IL-6 (R&D Systems, Minneapolis, MN, USA), PTX3 (R&D Systems), OPN (R&D Systems), OC (Metra/Quidel, CA, USA), OPG (BioVendor, Brno, Czech Republic), FGF-23 (Immunotopics Int., San Clemente, CA, USA), fetuin A (BioVendor). Total antioxidant capacity of plasma was measured as the ability of plasma to reduce Fe^3+^ to Fe^2+^ (ferric reducing ability of plasma – FRAP), according to Benzie’s method [16]. Radical scavenging capacity of plasma was estimated by DPPH radical scavenging assay as described elsewhere [17]. Ferric reducing ability of ascorbate in plasma (FRASC) was measured spectrophotometrically.

### Histology

Small fragments of radial artery wall, approx. 5 × 2 mm in size, were collected during the first creation of arteriovenous fistula for hemodialysis access. The samples were fixed overnight in 10 % phosphate-buffered formalin, then rinsed in PBS and soaked in 30 % sucrose. The material was snap-frozen and tissue blocks were positioned in a cryostat to allow cutting sections in a longitudinal plane of the vessel encompassing the entire thickness of the vascular wall. Serial 10 μm-thick cryosections were cut and thaw-mounted on poly-L-lysine coated slides. Sections were stained routinely with Mayer’s haematoxylin and eosin (HE) for general morphology as well as with von Kossa method and alizarin red for calcifications. The stained sections were examined under Olympus BX-50 microscope (Olympus, Tokyo, Japan) in brightfield mode and the images were acquired using Olympus DP-71 digital CCD camera controlled by Olympus AnalySIS FIVE software. The advancement of vascular calcification was semiquantitatively evaluated in von Kossa and alizarin red-stained sections by two independent observers. The degree of mineralization was classified according to the following scale: 0 - no mineral content, 1 - a few small dispersed concretions, 2 - numerous small dispersed concretions, 3 - larger granular concretions, 4 - large areas occupied by fused mineral deposits.

**Fig. 1:**
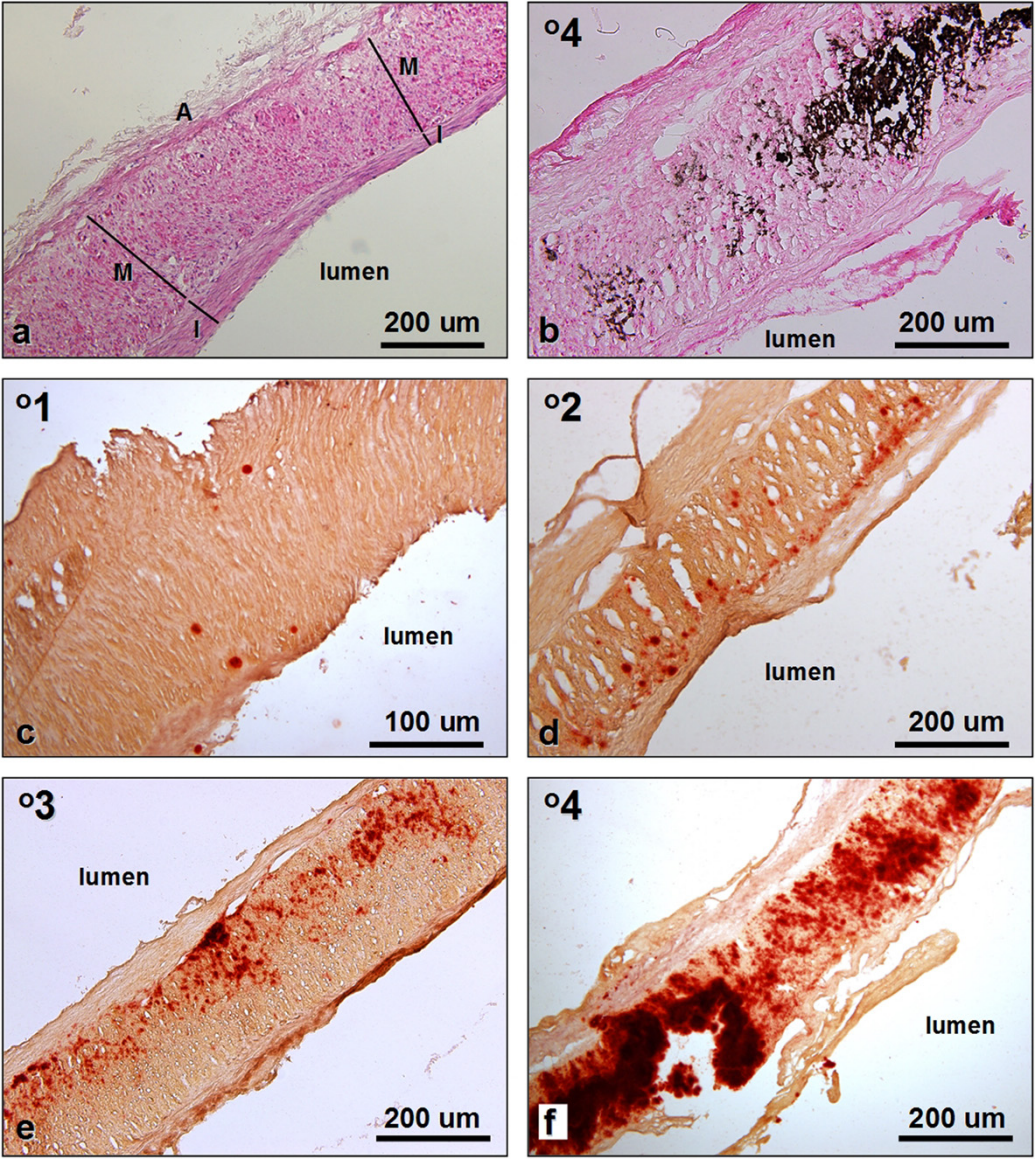
Histology of the radial artery samples. **a**. Morphology of the routinely (HE) stained vessel showing intimal thickening (I) and no detectable mineral content. I, intima; M, media; A, adventitia; for I/M ratio calculations thicknesses of intima and media were measured in two locations. **b**. Advanced calcification in artery stained with von Kossa method. **c-f**. Radial artery calcifications of various grades in samples stained with alizarin red

Since intimal thickening was postulated to be a marker of vascular pathology, the thicknesses of intima and media were measured in two distinct locations in sections stained with HE and mean intima to media (I/M) ratio was calculated (Fig. 1a).

The reproducibility of the morphological analysis was confirmed by Bland-Altman method and by calculating intraclass correlation coefficient (ICC) which was 0.88.

### Statistical methods

The data are expressed as the number of patients (percentage of the group) for categories and as mean ± SD or median (lower-upper quartile) for continuous variables, depending on the distribution, as assessed by the Shapiro-Wilk test. Chi-squared test was used to analyze contingency tables. Student *t*-test or Mann–Whitney test were used for simple comparisons between the groups. For logistic regression models, odds ratios (OR) were reported with 95 % confidence intervals (95 % CI). In order to assess the relationship between CCA-IMT and the degree of vascular calcification, one-way ANOVA with trend analysis was used. Simple correlations were analyzed with Spearman rank correlation coefficient. To calculate the multiple linear regression models, right-skewed variables were log-transformed; beta +/− standard error (SE) are reported. Survival times were calculated from the creation of arteriovenous fistula to death, or censored at the end of the follow-up, or renal transplantation. They were analysed using Kaplan-Meier method and compared with log-rank test. Also, simple and multiple Cox regression was calculated and hazard ratios (HR) were reported with 95%CI. All multiple regression models were adjusted for Framingham risk score and dialysis status at the time of surgery. The tests were two-tailed and the results were considered significant at *P* ≤ 0.05. Statistica 10 software (StatSoft, Tulsa, OK, USA) was used for the computations.

## Results

### Characteristics of the study group

Clinical characteristics of the patients and the results of laboratory tests are presented in Table 1. We also compared these parameters in patients with CCA-IMT above and below the median value.

**Table 1:** Clinical and laboratory characteristics of the studied group at the beginning of the study and its comparison in groups with low CCA-IMT (below median) and high CCA-IMT (above median)

	Total cohort (N = 59)	CCA-IMT ≤ median (N = 30)	CCA-IMT > median (N = 29)	*P*
Age, years	61+/−16	50+/−16	70+/−11	<0.001
Men, N (%)	38 (64)	18 (60)	20 (69)	0.5
Hemodialysed patients, N (%)	36 (61)	17 (57)	19 (66)	0.5
Dialysis therapy duration, months^a^	7 (2–37)	7 (1–37)	6 (1–32)	0.7
BMI, kg/m^2^	26.2+/−5.7	25.5+/−3.6	27.3+/−7.8	0.9
Diabetes, N (%)	19 (32)	5 (17)	12 (41)	0.036
Ischemic heart disease, N (%)	29 (49)	11 (37)	18 (62)	0.051
Heart failure, N (%)	13 (22)	3 (10)	10 (34)	0.023
Active smoking, N (%)	17 (29)	6 (20)	11 (38)	0.1
Hypertension, N (%)	26 (44)	15 (50)	11 (38)	0.4
Framingham risk score, points	8 (5–9)	5 (2–8)	9 (6–12)	<0.001
Serum creatinine, μmol/l	452 (326–527)	462 (408–527)	410 (32–512)	0.2
eGFR (MDRD), ml/min/1.73 m^2b^	14 (10–15)	10 (6–11)	10 (9–13)	0.6
Fasting glucose, mmol/l	5.0 (4.6-6.1)	4.8 (4.4-5.1)	5.5 (4.8-8.4)	0.022
Ca x Pi, mmol^2^/l^2^	3.05 (2.87-3.86)	3.57 (2.86-4.16)	2.98 (2.88-3.60)	0.2
iPTH, pg/ml	260 (180–453)	266 (204–414)	211 (102–403)	0.5
IL-6, pg/ml	4.20 (2.16-7.52)	2.63 (1.96-6.05)	5.17 (2.94-7.52)	0.3
CRP, mg/l	6.91 (2.97-19.00)	5.15 (2.14-9.73)	8.80 (2.11-22.4)	0.7
PTX 3, ng/ml	1.24 (0.70-2.51)	1.00 (0.68-1.71)	1.55 (0.74-2.26)	0.3
OPN, ng/ml	307 (212–513)	320 (207–588)	281 (217–352)	0.4
OPG, pmol/l	7.55 (4.36-12.00)	5.03 (2.62-9.36)	9.39 (6.76-12.38)	0.031
OC, ng/ml	41.8 (29.0-67.6)	48.7 (35.1-72.7)	33.6 (27.1-42.8)	0.053
Fetuin A	0.245+/−0.052	0.244+/−0.047	0.246+/−0.058	0.6
FGF 23	1013 (416–2529)	1006 (446–1220)	1082 (465–2618)	0.5
FRAP, mM/l	0.77 (0.55-1.14)	0.87 (0.52-1.06)	0.74 (0.57-0.91)	0.9
FRASC, μM/l	50.4+/−13.2	51.2+/−11.7	47.3+/−10.6	0.4
DPPH, %	39.9 (34.4-49.4)	40.5 (37.5-48.1)	36.4 (31.9-44.1)	0.07
CCA-IMT, mm	0.93+/−0.15	0.80+/−0.09	1.05+/−0.08	-
Atherosclerotic plaques in common carotid artery, N (%)	15 (25)	3 (10)	12 (41)	0.006

The median value of CCA-IMT equal to 0.925 mm

^a^data for hemodialysed patients

^b^data for predialysis patients

Patients with CCA-IMT above the median value were older, they had higher prevalence of diabetes and heart failure and higher fasting blood glucose and osteoprotegerin. Consequently, the Framingham risk score was also higher in these patients.

### Histological findings

Routine histology (HE staining) revealed the structure of radial artery with characteristic intimal thickening in the vast majority of the examined samples (Fig. 1a). The relative thicknesses of the intima (I/M ratios) varied between the samples (Table 2). The intimal thickening was mostly due to the presence of smooth muscle cells. Atheromatous lesions were not observed. Basophilic deposits were visible in routinely stained sections of highly calcified vessels (Fig. 1b). Mineralization of the arterial wall was visualized by both von Kossa method and alizarin red staining (Fig. 1 b-f). However, von Kossa staining detected calcifications in 17 (29 %) specimens, while alizarin red demonstrated minerals in 34 samples (58 %, Table 2). Large and medium-sized mineral deposits were successfully visualized by both methods, but von Kossa staining failed to demonstrate some finest deposits (grade 1 and 2). Moreover, scores calculated on the basis of von Kossa method were in most cases lower than those determined by alizarin red staining. Since alizarin red showed higher sensitivity, we adopted this method for further analysis and for comparison with the clinical and biochemical data [7].

**Table 2:** Histological parameters of radial arteries in the studied patients

Radial artery calcification	CKD stage 5 patients (N = 59)
Von Kossa staining:	
Grade 0	42 (71 %)
Grade 1	2 (3 %)
Grade 2	4 (7 %)
Grade 3	4 (7 %)
Grade 4	7 (12 %)
Alizarin red staining:	
Grade 0	25 (42 %)
Grade 1	12 (20 %)
Grade 2	5 (9 %)
Grade 3	9 (15 %)
Grade 4	8 (14 %)
*Intima* thickness, μm	60.7 (41.4-79.1)
*Media* thickness, μm	392 (332–461)
*Intima/media* ratio	0.15 (0.12-0.23)

Number of patients (%) or mean (range)

The mineral deposits were found most frequently in the vascular media. They presented different degrees of advancement (Fig. 1c-f). Smaller minerals were preferentially seen close to the inner and outer elastic laminae (Fig. 1d,e).

### Correlates of CCA-IMT

Among the studied parameters, fasting blood glucose (*r* = 0.37; *P* = 0.014), osteoprotegerin (*r* = 0.49; *P* = 0.002), pentraxin 3 (*r* = 0.36; *P* = 0.027) and FRS (*r* = 0.47; *P* = 0.001) were significantly correlated with CCA-IMT in simple analysis. We noted no significant correlations of CCA-IMT with other bone markers studied (osteopontin, osteocalcin, FGF 23, fetuin A), or with other inflammatory markers (CRP, IL-6) and with indicators of oxidative stress (FRAP, FRASC, DPPH scavenging). In multiple regression analysis, only log(OPG) significantly predicted CCA-IMT (beta = 0.41 ± 0.16, *P* = 0.017), independently of fasting blood glucose, PTX3, FRS and dialysis status.

Patients with atherosclerotic plaques detected by ultrasonography of common carotid artery had higher OPG [10.40 (7.70-14.15) vs 5.93 (3.56-8.58) pmol/l; *P* = 0.008]. As expected, the presence of plaques was associated with significantly higher CCA-IMT (1.04 ± 0.11 vs 0.86 ± 0.13 mm; *P* < 0.001).

**Fig. 2:**
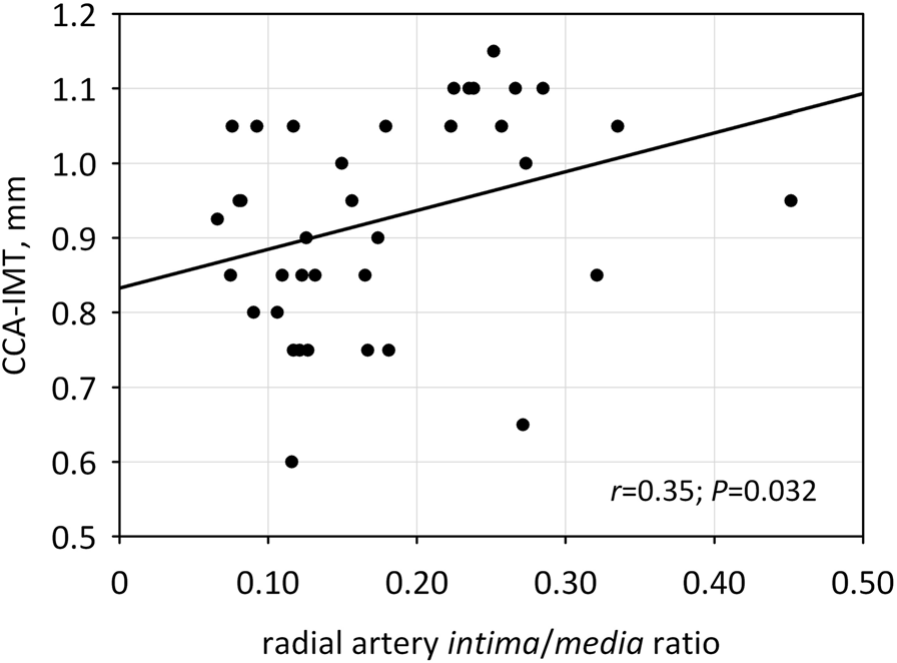
The correlation between common carotid artery intima-media thickness (CCA-IMT) and radial artery *intima/media* ratio. Pearson correlation coefficient with p-value is shown in the graph

The *intima/media* ratio measured histologically in radial artery samples positively correlated with CCA-IMT (*r* = 0.35; *P* = 0.032) (Fig. 2) and FRASC (*r* = 0.40; *P* = 0.008).

### Association of CCA-IMT with vascular calcifications

**Fig. 3:**
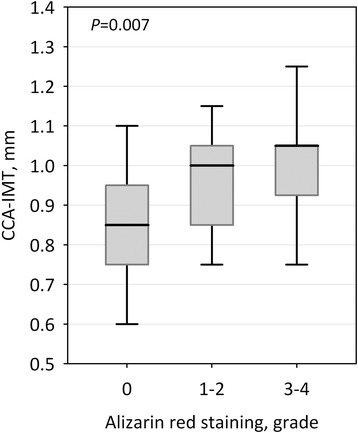
Common carotid artery intima-media thickness (CCA-IMT) values in patients without radial artery calcifications (alizarin red staining grade 0), with mild calcifications (grade 1–2) and with severe calcifications (grade 3–4). Data are shown as median, interquartile range (box) and range (whiskers); p-value for trend is shown in the graph

Patients with radial artery calcifications detected by histology had higher CCA-IMT (0.98 ± 0.13 vs 0.86 ± 0.14 mm; *P* = 0.006). In patients with CCA-IMT above the median value (0.925 mm), the incidence of vascular calcifications was twice as high as in patients with lower CCA-IMT (79 % vs 37 % of patients; *P* = 0.033). Also, higher CCA-IMT values were associated with more severe calcifications (one way ANOVA, F_2,41_ = 4.42; *P* = 0.018; *P* for trend =0.007; Fig. 3). In logistic regression analysis, CCA-IMT value significantly predicted vascular calcifications, independently of dialysis status, Framingham risk score, CRP and Ca x Pi (*P* = 0.024; Table 3). Similarly, the presence of atherosclerotic plaques in common carotid artery detected ultrasonographically was an independent positive predictor of radial artery calcifications (OR 7.39; 95 % CI 1.03-52.9; *P* = 0.037).

**Table 3:** Logistic regression models to study the association between CCA-IMT and radial artery calcifications

Predictor variable	OR for radial artery calcifications (95 % CI)		
	Simple model	Multiple model 1	Multiple model 2
CCA-IMT, 0.1 mm	1.90 (1.13-3.19)	1.83 (1.02-3.30)	2.19 (1.08-4.45)
Framingham risk score, points	-	1.03 (0.87-1.22)	1.06 (0.86-1.29)
CRP, mg/l	-	-	1.03 (0.97-1.09)
Ca x Pi, mmol^2^/l^2^	-	-	1.71 (0.54-5.41)

Multiple models 1 and 2 were additionally adjusted for dialysis status

### Association of radial artery calcification and CCA-IMT with mortality

During 3-year follow-up period, 20 (34 %) patients died, mostly (18 patients, 90 %) due to cardiovascular causes, including 7 deaths due to myocardial infarction, 3 deaths due to cerebral stroke and 8 due to heart failure. Two deaths were due to neoplasms. Seven patients underwent renal transplantation. The median observation period was 36 (22–36) months; overall, we assessed 140 patient-years. Lower quartile of survival was 24 months, median survival was not reached.

**Fig. 4:**
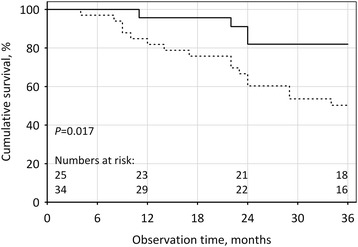
Overall survival in patients with (dashed line) and without (solid line) radial artery calcifications. Numbers at risk at the beginning and at the end of 1^st^, 2^nd^ and 3^rd^ year of observation are shown at the bottom of the graph

Among 34 patients with radial artery calcifications, 16 (47 %) died, including 15 due to cardiovascular causes. In contrast, there were only 4 deaths (16 %, including 3 cardiovascular) among 25 patients without calcifications (*P* = 0.017; Fig. 4). In multiple Cox regression, the presence of radial artery calcifications was a significant predictor of all-cause and cardiovascular mortality, independent of dialysis status and Framingham risk score (HR3.16; 95% CI 1.03-9.64; *P* = 0.043 and HR 3.97; 95% CI 1.13-14.00; *P* = 0.032, respectively).

CCA-IMT above the median value was also associated with more frequent cardiovascular deaths [13 (45 %) vs 5 (17 %) patients, *P* = 0.020], however, multiple Cox regression analysis did not confirm this result.

## Discussion

This is the first study of vascular calcification aimed at assessing the correlation between the results of a non-invasive imaging technique (ultrasound) and histological findings in the living patients, since the microscopic studies of calcified vessels have been mostly performed on autopsy specimens [18, 19]. The histological examination of the vessels collected on the occasion of a routine medical procedure (creation of arteriovenous fistula for hemodialysis access) enables to assess not only the severity of calcifications, but also their intimal or medial location. These locations represent two distinct processes of vascular calcification: the intimal calcification is mostly associated with advancement of atherosclerosis and has inflammatory background, whereas medial arterial calcification (MAC) develops independently of atherosclerosis and resembles physiological mineralization, e.g. that of bone tissue [5, 6, 11, 19, 20]. Such calcification is commonly observed in diabetes and end stage renal disease [7, 13, 21–23]. MAC lesions are characterized by calcific deposits between smooth muscle cells and elastic lamine within the media of medium-sized and small muscular arteries and they do not cause luminal narrowing. Atherosclerosis is characterized by atheromatous plaques containing lipids, inflammatory cells and matrix components in the intima of large and medium-sized arteries [5, 11]. In the present study, mineral deposits were found almost entirely in the vascular media of radial arteries. Only very scanty mineral deposits were occasionally seen in the vascular intima and no atheromas were observed. Our findings demonstrate the presence of MAC type calcification in radial arteries of CKD patients and confirm results of others [19, 24].

Histological studies of vascular calcification usually employ von Kossa method or alizarin red staining to demonstrate mineral deposits. We compared both procedures. In our study, alizarin red staining showed considerably higher sensitivity. Hence, we strongly suggest the use of alizarin red instead of von Kossa staining for histological detection of vascular calcification in samples of human arteries.

Currently, vascular calcification can be clinically assessed by multislice spiral CT (MSCT) and electron beam CT (EBCT). These methods are associated with high exposure to X-ray radiation and they are of high cost. Moreover, they are not widely available and can be performed only in specialized diagnostic centers. X-ray imaging allows to identify extensively calcified lesions and does not distinguish between the intimal and medial calcifications. As recently demonstrated, ultrasonography [21, 24] can be used to detect both types of calcifications, since it allows to distinguish different layers of the arterial wall. It also shows higher sensitivity, detecting vascular calcifications in higher proportion of cases as compared with standard X-ray imaging (46 % vs. 21 %). The authors postulate that ultrasonography could potentially provide an alternative method for the diagnosis of MAC but probably only in large vessels, such as femoral arteries [21].

The predominant type of arterial calcification in CKD patients still seems to be a matter of controversy. A study by Coll’s group [24] using ultrasound to determine the location of mineral lesions in the arteries of dialysed renal patients demonstrated that vascular calcification of capacitance arteries was associated with the presence of atherosclerosis. They studied the presence of vascular calcifications and atheromatous plaques in carotid, femoral and brachial arteries and found that the most common type of vascular calcification was linear calcification of the intima associated with the presence of plaques. Linear intimal calcification probably corresponds to the calcification of internal elastic lamina, demonstrated histologically in coronary arteries [25].

Coll and coworkers [24] also concluded that the absence of carotid plaque was a protective factor for development of linear calcification. In arteries with a low prevalence of plaques linear calcification was rarely observed (7.5 % of patients on dialysis). These results seem to indicate that the predominant type of vascular calcification of large arteries in patients on dialysis is associated with the presence of atherosclerosis.

CCA-IMT was shown to be associated with cardiovascular risk factors, prevalent cardiovascular disease, atherosclerosis and vascular calcification in peripheral arteries [26]. Earlier reports confirmed the association of CCA-IMT with coronary artery calcium score (CACS) and cardiovascular disease not only in patients with chronic kidney disease [10] but also in patients with diabetes type 2 [22] and rheumatoid arthritis [27]. We demonstrated that the presence of abnormal carotid IMT (>0.925 mm) led to two times more prevalent vascular calcification risk. The presence of sonographically detected atherosclerotic plaques in common carotid artery was also an independent positive predictor of radial artery calcifications. As expected, in our study the presence of plaques was associated with significantly higher CCA-IMT. Similarly, in a study by Kurnatowska et al. [10], coronary artery calcification (CAC) occurred in 70.2 % of dialysis patients and it was significantly associated with CCA-IMT and with the thickness of atherosclerotic plaques. Carotid plaque evaluation may have important clinical implications by identifying a subgroup of high cardiovascular risk in asymptomatic diabetic and CKD patients. Thus, measurement of CCA-IMT might indirectly indicate an increased risk of MAC and could serve as noninvasive method for assessing overall cardiovascular risk in this population.

Our study confirmed the relationship between CCA-IMT and cardiovascular risk factors in CKD patients [4, 7, 20, 21, 26, 28]. However, its results show that not only the classical risk factors can affect the intima-media thickness. Although patients with CCA-IMT above the median value were older, had higher prevalence for diabetes, heart failure and higher concentrations of fasting blood glucose and osteoprotegerin, in multiple regression analysis, only log(OPG) was an independent predictor for IMT. The association of CCA-IMT with OPG level was also observed by us in CKD patients on peritoneal dialysis [29]. In another study, both increased CACS as well as CCA-IMT positively correlated with baseline and follow-up serum OPG. The patients who died had significantly higher baseline CACS and serum OPG [10]. Osteoprotegerin (OPG), a member of the tumor necrosis factor receptor family, has been identified as a regulator of bone resorption and inhibitor of vascular calcification. Increased levels of OPG in CKD patients with vascular calcifications can represent a response to mineral disorders and constitute a compensatory mechanism.

Our study confirmed earlier reports demonstrating high cardiovascular mortality associated with vascular calcification not only in dialyzed patients [20, 30, 31] but also in CKD patients not yet on dialysis [23]. The presence of radial artery calcifications was a significant predictor of mortality, independent of dialysis status and Framingham risk score.

In summary, our results indicate that both medial arterial calcification and intimal atherosclerotic changes frequently coexist in CKD patients. Elevated CCA-IMT is associated with higher incidence and severity of medial arterial calcification which in turn predicts adverse clinical outcomes and higher mortality in such patients.

## Conclusions

In CKD patients, sonographic examination of CCA-IMT can be used as an early, non-invasive screening method assessing the risk of arterial medial calcification and identifying patients at high risk of cardiovascular disease. That would allow for early prevention and reduction of mortality in these patients.

## Abbreviations

BMI: Body mass index
Ca: Total calcium
CAD: Coronary artery disease
CACS: Coronary artery calcium score
CCA-IMT: Common carotid artery intima-media thickness
CKD: Chronic kidney disease
hsCRP: High-sensitivity C-reactive protein
DPPH: 2,2-diphenyl-1-picrylhydrazyl
EBCT: Electron-beam computed tomography
eGFR: Estimated glomerular filtration rate
FGF-23: Fibroblast growth factor-23
FRAP: Ferric reducing ability of plasma
FRASC: Ferric reducing ability of ascorbate in plasma
FRS: Framingham Risk Score
HE: Haematoxylin and eosin
HOMA-IR: Homeostasis Model of Assessment - Insulin Resistance
IMT: Intima-media thickness
ICC: Intraclass correlation coefficient
I/M ratio: Intima to media ratio
MAC: Medial arterial calcification
MDRD: Modification of Diet in Renal Disease
MSCT: Multi-slice (spiral) computed tomography
OC: Osteocalcin
OPG: Osteoprotegerin
OPN: Osteopontin
iPTH: Intact parathormone
IL-6: Interleukin-6
Pi: Phosphate
PTX 3: Pentraxin 3
